# Survival differences by ethnicity among patients diagnosed with bladder cancer

**DOI:** 10.1186/s12967-020-02668-3

**Published:** 2020-12-14

**Authors:** Boda Guo, Ming Liu

**Affiliations:** 1 Department of Urology, Beijing Hospital, National Center of Gerontology; Institute of Geriatric Medicine, Chinese Academy of Medical Sciences, Beijing, 100730 China; 2 Graduate School of Peking, Union Medical College, Beijing, 100730 China

Dear Editor,

We read the study by Fang et al. [1] with great interest and would like to congratulate the authors for their superb study. The authors compared the oncologic outcomes of bladder cancer (BC) of different ethnicities, which may aid in clinical decision-making. As such, there are a few points that we would like to bring up.

1. Their study included patients with primary BC but did not exclude multiple primary cancers. However, another SEER-based study showed BC patients were at a significantly higher risk of developing a second malignancy, which may affect patients’ survival [2].
2. BC surgery in the SEER database includes transurethral resection of bladder, partial cystectomy and complete cystectomy, etc. It would be more meaningful to stratify patients according to the type of surgery that they received; in this way, readers can easily see which surgical treatment improves BC patient’s survival better.
3. Some of the patients included in the analysis received radiotherapy or chemotherapy. These two types of treatment should be taken into consideration since prior studies showed that both radiotherapy and chemotherapy hold great potential in the management of BC patients [3, 4].

## Data Availability

Not applicable.

## Abbreviation

BC: Bladder cancer
